# Homeostatic Signaling by Cell–Cell Junctions and Its Dysregulation during Cancer Progression

**DOI:** 10.3390/jcm5020026

**Published:** 2016-02-17

**Authors:** Yang Yu, Randolph C. Elble

**Affiliations:** 1 Department of Nature Medicine, Tianjin Medical University School of Pharmacy, Tianjin 300070, China; yuyang@tmu.edu.cn; 2 Department of Pharmacology, Southern Illinois University School of Medicine, Springfield, IL 62794, USA; 3 Simmons Cancer Institute, Southern Illinois University School of Medicine, Springfield, IL 62794, USA

**Keywords:** E-cadherin, EMT, dissemination, breast cancer, human mammary epithelial cells, adherens junctions, tight junctions, claudins, PLEKHA7, miR30b, cancer stem cells

## Abstract

The transition of sessile epithelial cells to a migratory, mesenchymal phenotype is essential for metazoan development and tissue repair, but this program is exploited by tumor cells in order to escape the confines of the primary organ site, evade immunosurveillance, and resist chemo-radiation. In addition, epithelial-to-mesenchymal transition (EMT) confers stem-like properties that increase efficiency of colonization of distant organs. This review evaluates the role of cell–cell junctions in suppressing EMT and maintaining a quiescent epithelium. We discuss the conflicting data on junctional signaling in cancer and recent developments that resolve some of these conflicts. We focus on evidence from breast cancer, but include other organ sites where appropriate. Current and potential strategies for inhibition of EMT are discussed.

## 1. Breast Cancer

Breast cancer is the second most common cancer among women in the United States. Major achievements in breast cancer research in recent decades have led to a remarkable improvement in the five-year survival and quality of life. However, metastatic breast cancer remains a clinical challenge. For breast cancer patients of stages 0-III (*in situ*), the five-year survival is up to 52%; however, for stage IV (metastatic) breast cancer patients, the percentage drops to 16. Because of the high death rate and lack of effective therapies, prevention and treatment of metastasis is a major focus of cancer research.

Metastasis is a molecularly complex process involving a series of cellular events collectively known as the invasion-metastasis cascade [1,2,3,4]. For a cancer cell to form a metastatic secondary tumor, it needs to overcome a variety of challenges throughout its journey and at the secondary site. Numerous studies in the past dozen years have established that the epithelial-to-mesenchymal transition (EMT) program is frequently reactivated in invasive cancer cells and confers metastatic potential [5,6].

## 2. Epithelial-to-Mesenchymal Transition (EMT), Tumor Invasion, and Metastasis

### 2.1. Physiological Significance

EMT is characterized by loss of cell–cell junctions, loss of apical-basal and planar polarity, cytoskeletal remodeling, morphological changes, and acquisition of migratory and invasive capabilities [5].This process involves a shift in regulation of a cohort of genes, some of which are downregulated and others of which are upregulated (reviewed in [7]). EMT and the reciprocal process, mesenchymal-to-epithelial transition (MET), are important developmental and physiological programs. In higher vertebrates, EMT generates a migratory mesenchyme that invades distant regions of the embryo and re-differentiates to form new tissues and organs [8,9]. The capacity of adult epithelial cells to undergo EMT identical to that of embryonic cells was first demonstrated by Greenburg and Hay in 1982 using 3D collagen culture [10].

In adults, EMT is required for wound healing. In response to a sudden tear in epithelium, neighboring cells must undergo EMT to invade the gap then undergo MET to proliferate, refill the gap and re-establish cell–cell junctions anchored to the cytoskeleton. This was illustrated by Savagner who showed in mouse keratinocytes that epithelial cells lining a wound *in vivo* or *in vitro* developed high expression of the mesenchymal transcription factor Slug, lost desmosomes, and underwent EMT [8]. Wound healing was profoundly inhibited in skin explants from Slug-knockout mice. After wound closure, such cells presumably undergo MET to return to epithelial phenotype.

The EMT program is frequently “hijacked” by carcinomas during cancer progression [5,6,7,11]. The role of EMT as a crucial event leading to cancer cell invasion and metastasis has been reported in many solid tumor cancers including those of breast, prostate, pancreas, colon, and bladder [7,11,12,13,14,15,16]. EMT confers many features to cancer cells that favor the invasion-metastasis cascade, described below.

### 2.2. EMT Confers Invasiveness

During EMT, the loss of cell junction proteins, including both adherens junctions and tight junctions, allows epithelial cells to escape the bonds of neighboring cells and acquire the motility needed for metastasis [5,6]. Furthermore, the remodeling of cells of the cytoskeleton leads to significant morphological changes. The resulting elongated, fibroblast-like morphology of mesenchymal cells facilitates migration and breaching of the basement membrane. The penetration of this membrane is also promoted by the increased activity of matrix metalloproteases (MMPs) in post-EMT cells. These MMPs include MT1-MMP, MMP-2, -9, -12 and -13 [17].

### 2.3. EMT Confers Resistance to Anoikis

To survive outside the primary organ, cancer cells must become resistant to detachment-induced apoptosis, anoikis. Resistance to anoikis is a hallmark property of EMT. Derksen *et al.* reported that E-cadherin (E-cadherin) played an important role in sensitizing epithelial cells to anoikis. Knockout of E-cadherin in a p53-dominant-negative tumor cell model enhanced tumor metastasis, and the derived cultured cells were highly resistant to anoikis compared with E-cadherin-expressing control cells [18]. The role of EMT in anoikis resistance was further confirmed by Onder *et al.* using the immortalized human mammary epithelial cell line HMLE that had been induced to EMT by knockdown of E-cadherin [19]. They showed that anoikis resistance was partly due to the activation of the Wnt signaling pathway; and knockdown of β-catenin, an effector of this pathway, restored sensitivity to anoikis. Thus, β-catenin plays a pivotal role in EMT and cancer cell survival.

### 2.4. EMT Confers Stemness

One of the most exciting and paradigm-shifting developments in cancer biology in this century was the demonstration by Weinberg and co-workers that EMT confers stem cell properties to cancer cells, giving rise to cancer stem cells (CSCs), also known as tumor-initiating cells [11].This discovery was made possible by the earlier studies of Wicha’s lab, establishing that mammary epithelial cells contain a subpopulation with stem cell properties such as pluripotency and anoikis resistance; signature markers were later identified that would allow their isolation by flow cytometry and characterization by transcriptional profile [20]. Subsequent work showed that breast cancer cell lines contained subpopulations with the same properties and markers and that these cells were also enriched for tumor-initiating capability [21]. Here, the studies of CSCs began to converge with those of induced EMT. Mani *et al.* found that ectopic expression of EMT transcription factors in transformed human mammary epithelial cells (HMEC) resulted not only in expression of mesenchymal markers but also enrichment for expression of stem cell markers and behaviors such as migratory behavior, resistance to DNA-damaging agents, and ability to form colonies when suspended in non-adherent media or implanted into immunocompatible mice [9]. The congruency between these two phenotypes was further underlined by the demonstration that sorting immortalized HMEC or tumor cells for stem cell markers also enriches for mesenchymal markers. Others showed that breast cancer cell lines with a mesenchymal profile were also greatly enriched for CSC markers and behaviors [21]. Thus, a mutation that triggered EMT in a tumor would also confer CSC properties and facilitate colonization of distant organ sites [16].

### 2.5. EMT Confers Immunoresistance

Another advantage conferred upon the tumor cell by EMT is resistance to lysis mediated by cytotoxic T lymphocytes (CTL). Akalay *et al.* induced EMT in MCF7 breast cancer cells by ectopic expression of activated Snail transcription factor and tested their susceptibility to CTL [22,23]. They reported that EMT cells were resistant to synapse, with CTL and subsequent lysis. They noticed that the EMT cells had higher levels of autophagy and found that inhibition of autophagy by beclin knockdown restored sensitivity to CTL. Moreover, IHC of breast cancer specimens revealed a positive correlation between Snail expression and markers of autophagy. These properties would be expected to increase the survival rate of cancer cells both in circulation and at distant sites. More recently, it was discovered that EMT upregulates PD-L1 immune checkpoint ligand [24]. This may explain the reduction in immune synapse reported by Akalay. It is of therapeutic importance that downregulation of PD-L1 partly reversed the EMT.

### 2.6. Induction of EMT by Soluble Factors

Given the centrality of EMT in promoting metastasis, identifying approaches to suppress cancer cell EMT has attracted great interest. To that end, it is critical to understand how EMT is activated, executed and regulated in cancer cells. In normal tissues, EMT is set in motion by extracellular developmental or physiological cues such as morphogens (Wnt, Shh, and Notch), prostaglandins, and various cytokines (e.g., TGFβ and IL6) [25,26,27]. These substances are secreted by myofibroblasts, mesenchymal stem cells, senescent stromal cells, or immune cells in the microenvironment, and engage epithelial receptors [28,29]. Receptor ligation activates signaling cascades that converge ultimately on a suite of master transcription factors of the Snail, Twist, and Zeb families that activate mesenchymal programs and repress expression of junctional moorings; all potently repress E-cadherin [28].

How neoplastic epithelial cells avail themselves of this program is a matter of debate. It is important to realize that every cancer is an organism that has never existed before, and that this organism has the entire human genome at its disposal along with every genetic program encoded there and whatever new ones that random mutation and *in vivo* selection can bestow upon it. Thus, there may be good evidence in one case that a tumor cell at the primary site experienced a mutation that triggered EMT and allowed its descendants to metastasize, while in another case, microenvironmental influences may have allowed escape by inducing transient “partial EMT” in an otherwise nonmigratory cell. Indeed, the phenomenon of EMT in cancer was independently discovered by immunologists studying tumor cell interaction with the immune system [30]. Recently, Alkalay *et al.* showed that EMT could be potently induced in MCF7 cells by exposure to ever higher levels of the inflammatory cytokine TNF α [23]. The authors devised a system for quantifying completeness of EMT and found that TNF α produced a more complete EMT than one induced by Snail overexpression. Moreover, combined treatment with TGFβ and TNFα generated a claudin-low phenotype that has been linked to stem-like properties and a high probability of recurrence [31].

The occurrence of partial EMT is supported by the finding that IHC of tumor biopsies often reveals heterogeneity of EMT/MET marker expression, even in the same cell [25]. Conceptually, plasticity of differentiation better promotes tumor cell survival than being locked into EMT by mutational changes. A mesenchymal phenotype is advantageous until the tumor cell arrives in a distant organ. At that point, it will no longer be subject to the environmental cues that provoked EMT at the primary site and in transit. It no longer needs to “go” but instead must remember how to “grow” and generate a new tissue. The capacity to reverse the process by undergoing MET allows the interloper to re-express integrins that will interact with the new extracellular matrix, direct them to what will become the basal face of the cell, re-establish stable cell–cell interactions, and reactivate a proliferative program. Plasticity is intrinsic to EMT because EMT confers stem-like multipotency and tumor-initiating capabilities [4]. The ability of normal and transformed mammary epithelial cells to transition between these states has been well demonstrated [32,33,34]. The concept of EMT as a metastable condition with intermediate states was recently reviewed by Tam and Weinberg [35].

### 2.7. Structural Cues Suppress EMT

In addition to diffusible signals, epithelial sheets must also respond promptly to trauma. The cell-autonomous nature of the response can be readily observed by wounding an epithelial monolayer *in vitro*. Over the course of hours or days, cells bordering the wound break free from their neighbors and migrate individually into the vacant area. This behavior depends on an EMT induced by disruption of cell–cell junctions [8]. Indeed, Sarrio *et al.* have demonstrated that disrupting a differentiated HMEC monolayer with trypsin and seeding at low density is sufficient to produce EMT [36]. As cells then increase in density, they re-establish cell–cell junctions and undergo MET, and in the process become more resistant to disruption by trypsin. Moreover, cell dilution and differential trypsinization can be used to enrich for mesenchymal or epithelial subpopulations from the same cell line [37,38].

These observations underline the essential role of cell–cell junctions in maintaining epithelial homeostasis. They also indicate that EMT is a default pathway that is held in abeyance by structural continuity of cell–cell interactions. We will now consider the nature of cell–cell junctions, the mechanisms by which structural adhesion molecules prevent EMT, and how they are subverted during tumor progression.

## 3. Epithelial Cell–Cell Junctions Suppress EMT

**Figure 1:**
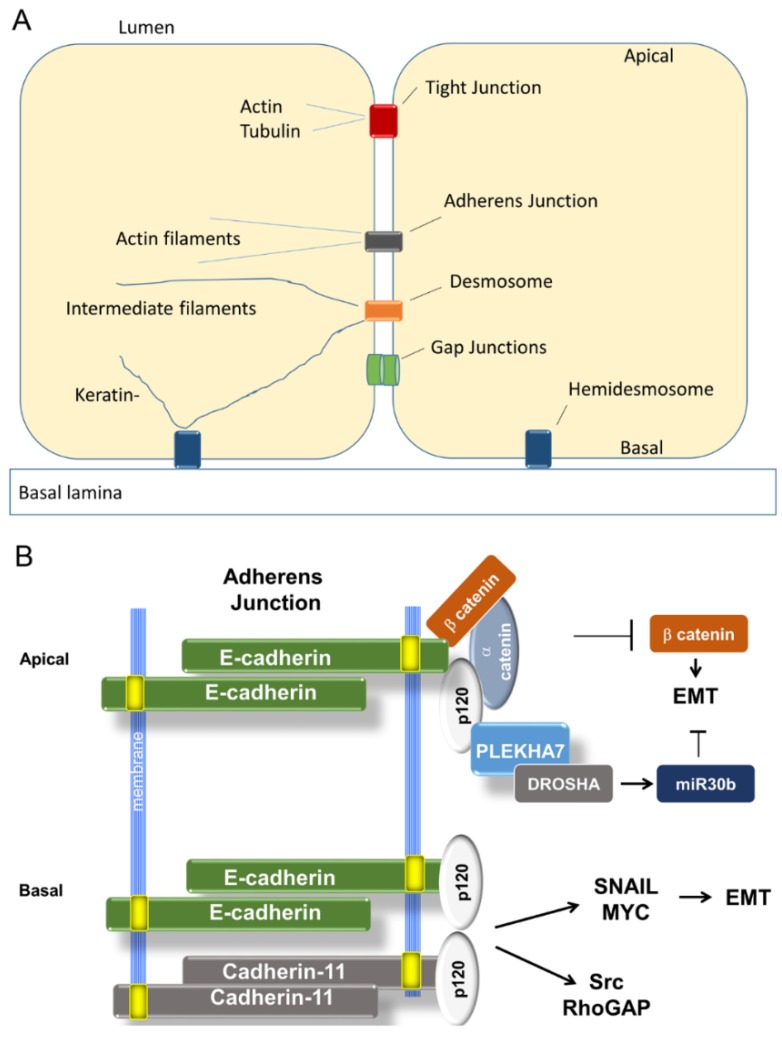
Epithelial junctions. (**A**) Generalized schematic showing types of cell–cell and cell-matrix attachments of simple columnar epithelium. Except for gap junctions, each complex is linked both to cytoskeletal elements and to its own signaling apparatus. (**B**) Regulation of EMT–MET (epithelial-to-mesenchymal transition–mesenchymal-to-epithelial transition) by Adherens Junctions, adapted from Kourtidis *et al.* [65]. Canonically, E-cadherin sequesters β-catenin and downregulates Twist. More recently, E-cadherin was found to participate in two functionally distinct complexes, an apical one that opposes EMT and a basal one that promotes it. Apically, it interacts with PLEKHA7 and a microRNA processing complex that suppresses proliferation and mesenchymal functions. However, in basolateral locations it actually promotes those functions. This may explain the inconsistent patterns of E-cadherin expression across breast cancer subtypes and the observation that it sometimes seems to promote invasion/metastasis rather than suppress it. For simplicity, E-cadherin and cadherin-11 are shown as monomers and cytoskeletal interactions are omitted.

Cell–cell junctions associated with cytoskeletal filaments are the basis of the tensile strength and structural integrity of epithelial tissues. A typical epithelial cell contains several major classes of functionally diverse cell–cell junctions: adherens junctions, tight junctions, desmosomes, and gap junctions (Figure 1A). Of these, only adherens junctions (AJ) and tight junctions (TJ) are known to play a necessary regulatory role in maintaining the epithelial program [19,39]. For more background on epithelial structure, see chapter 19 of Lodish *et al.* [40].

### 3.1. Adherens Junctions Suppress EMT

#### 3.1.1. Organization of Adherens Junctions (AJ)

When epithelial cells meet, one of the first cell–cell structures to form is adherens junctions, initiated by interaction of opposing cadherin ectodomains [22]. Cadherins are type I transmembrane adhesion proteins whose function is dependent on extracellular Ca^2+^. The classical cadherins are E-, N-, R- and P-cadherins (reviewed in [41]). Each classical cadherin contains a single transmembrane segment, a short *C*-terminal cytosolic domain and five extracellular cadherin domains that are important for Ca^2+^ binding and cell–cell adhesion. The distal cadherin domain is the most important domain for the conjunction with adjacent cells, while the *C*-terminal cytosolic domain is linked to the actin skeleton through adaptor proteins such as β-catenin, providing essential strong adhesion between cells.

Although structurally similar, these cadherins diverge functionally. N- and P-cadherins mediate weak, transient associations and are strongly associated with migratory behaviors. P-cadherin is expressed in progenitor cells and terminal end-buds in the developing mammary ducts [42]. It is only expressed in myoepithelium of the differentiated breast, but is upregulated in breast cancers.

N-cadherin is associated with mesenchymal cells, and the switch from E- to N-cadherin is a hallmark of EMT [5,43]. It is induced by the same mesenchymal transcription factors that repress E-cadherin [44]. This substitution allows epithelial cells to escape the homotypic interactions that bind them to adjacent epithelial cells and instead form weaker, transient adhesions with N-cadherin proffered by stromal fibroblasts [44,45]. The cytoplasmic tail of N-cadherin interacts with the cytoskeleton through the same catenins as E-cadherin. However, N-cadherin also interacts with neural cell adhesion molecule to promote focal adhesion formation and motility [46,47].Yamada and co-workers showed that these N-cadherin functions play a vital role in migration; knockdown of N-cadherin in post-EMT cells inhibited both cell–cell adhesion and migration in a 3D matrix [48]. The differing roles of N-cadherin and E-cadherin have also been probed in a revealing series of animal experiments. Kotb *et al.* found that substitution of N-cadherin for E-cadherin in mouse mammary glands by knock-in interfered with mammary development by causing premature involution and apoptosis of alveolar cells [49]. Remarkably, however, suppression of apoptosis by p53 inactivation allowed normal development and lactation. The mice later developed fibrocystic changes and eventually malignancy. Thus, N-cadherin is capable of providing cell–cell adhesion function to epithelial cells but fails at homeostatic signaling.

#### 3.1.2. Loss of E-Cadherin in Cancer and EMT

E-cadherin has long been of interest to cancer cell biologists because its loss is associated with increased tumor aggressiveness. In a pioneering study, Oka *et al.* reported that 86% of breast cancer metastases lacked junctional E-cadherin [50]. This correlation was later upheld in other cancer metastases [51,52]. Experimentally, restoration of E-cadherin to invasive cancer cell lines was seen to suppress invasion and partly restore epithelial features, while knockout in p53-deficient mice produced invasive lobular carcinoma [18,53]. The loss of E-cadherin was associated with loss of other epithelial markers, consistent with EMT. Downregulation of E-cadherin in breast cancer is associated with aberrant DNA methylation of the CDH1 gene promoter and/or repression by mesenchymal transcription factors that are known to cause promoter methylation [54,55].

These transcription factors, including Snail, Slug, and Zeb2, bind to and repress the E-cadherin promoter in response to numerous extracellular cues associated with EMT, such as Wnt, TGFβ, and IL6 [35]. In addition, they repress promoters of tight junctional and desmosomal cell–cell adhesion molecules [28]. Snail, Slug, Twist1, Zeb1, and Zeb2 are frequently upregulated or amplified in invasive breast cancers and are associated with poor prognosis [56,57,58]. Upregulation of Snail and Twist and downregulation of E-cadherin was detected by IHC at the invasive front of parathyroid tumors [59]. EMT at the invasive front has been reported for a variety of solid tumors, suggesting that EMT is frequently employed to penetrate the basement membrane [60,61,62].

In quiescent epithelium, the cytoplasmic tail of E-cadherin associates with a large complex of proteins linked to the actin cytoskeleton (Figure 1) [22]. Among them are three catenins, α, β, and p120, all of which are signal transducers in addition to their structural functions; all are required for AJ assembly [63]. Knockdown of p120-catenin destabilizes E-cadherin and prevents its surface localization [64].

The role of E-cadherin in signaling cell–cell adhesion to the nucleus and maintaining a homeostatic transcriptional program was unraveled by Onder *et al.* [19]. They found that lentiviral knockdown of E-cadherin (shE-cadherin) was sufficient to trigger EMT in immortalized HMEC and confer metastastic potential to transformed cells. On the other hand, a dominant-negative E-cadherin construct lacking the ectodomain reduced cell clustering but was unable to cause EMT, thus implicating the cytoplasmic tail.

To explain the EMT, the investigators posited that E-cadherin might sequester β-catenin, which could otherwise enter the nucleus, complex with TCF transcription factors, and activate expression of mesenchymal transcription factors, Myc, and other migratory and proliferative factors [66]. Activating or stabilizing mutations in β-catenin are common in breast cancers, and β-catenin had previously been associated with EMT and metastatic behavior [67]. To test the role of β-catenin in shE-cadherin-induced EMT, they knocked down its expression and found that the EMT was partly reversed, and metastasis was greatly inhibited. To determine whether β-catenin activation would be sufficient for EMT, they transduced a constitutively active mutant. It was unable to induce EMT, implying that β catenin activation is necessary but not sufficient for EMT. Subsequent work revealed that mesenchymal transcription factor Twist was upregulated by shE-cadherin and that knockdown of Twist partly reversed EMT. The mechanism for E-cadherin regulation of Twist remains unknown. However, we note that Twist is induced by multiple signaling pathways, including STAT3, AKT, Ras, and Wnt [28]. E-cadherin interacts with EGFR, an activator of Ras, and mutations in the E-Cadherin extracellular domain or disruption of AJ have been found to upregulate Ras [68,69]. Whether increased Ras signaling is responsible for Twist upregulation in response to E-cadherin knockdown remains to be tested. These results suggest that the E-cadherin cytoplasmic domain maintains epithelial homeostasis by multiple mechanisms, not all of which have been defined, and compromise of this function is a major driver in the invasion-metastasis cascade.

#### 3.1.3. Could E-Cadherin Abet Dissemination?

However, this simple model may not be the whole story. To test the role of E-cadherin in invasiveness, Shamir *et al.* devised a 3D-Matrigel model for cell dissemination [70]. Mammary explants from transgenic mice with inducible deletion of E-cadherin or expression of Twist1 were suspended in matrix, and cell behavior was tracked over time. Organoids were seeded and allowed to form cysts. While deletion of E-cadherin disrupted cell–cell junctions, it did not result in dissemination. In contrast, induction of Twist1 caused rapid dissemination and, surprisingly, the emigrating cells maintained surface expression of E-cadherin and β-catenin. Despite abundant evidence that Twist1 represses E-cadherin in other systems, here RNA expression profiles revealed little change in expression of epithelial cadherins, keratins, or other markers, though there was downregulation at the protein level. Still more unexpectedly, attenuation of E-cadherin in these cells actually inhibited dissemination. Although it may be argued that the system is artificial, the results could not be easily reconciled with the previously accepted model.

#### 3.1.4. A Tale of Two E-cadherins

Another recent landmark study provides some illumination. The investigators sought to explain the paradoxical observation that, depending on the presence of E-cadherin, p120 can promote either epithelial junctional integrity or EMT and anchorage-independent growth of breast cancer cells [71]. Key to resolving the paradox was the discovery by Kourtidis *et al.*, using proteomic methods, that E-cadherin and p120 exist in two spatially and functionally distinct complexes at cell–cell junctions, one apical and the other basal [65]. The apical complex contains p120 partner PLEKHA7 which tethers E-cadherin and p120 to proteins required for maturation of microRNAs that suppress EMT. Intact, this complex was tumor suppressive. The basal complex lacked this microprocessor association and instead promoted growth by activating src, Rho-GAP, and expression of Snail, Myc, and cyclin D. In addition, a new cadherin, cadherin-11, was recruited into the basal complex by p120, and mesenchymal functions were dependent on its presence. Knockdown of PLEKHA7 disrupted apical localization of E-cadherin and p120, and processing of tumor-suppressive microRNAs was misregulated. Furthermore, these cells became anoikis-resistant. Importantly, the last was dependent on continued expression of E-cadherin and p120 at basolateral junctions. These findings could explain why in the Shamir 3D system E-cadherin expression was necessary for dissemination. Presumably, the basolateral complex was needed to activate Snail. They also beg the question whether Twist1 disrupts the PLEKHA7 apical complex, leaving the basolateral signaling hub intact. Thus, it seems that the route to invasiveness may either downregulate E-cadherin or co-opt it.

#### 3.1.5. R-Cadherin

In addition to E-cadherin, several laboratories have reported that R-cadherin contributes to epithelial differentiation and that its knockdown similarly leads to EMT [72]. With breast cancer progression, R-cadherin is downregulated more consistently than E-cadherin. How the two coordinate their functions remains to be established.

### 3.2. Tight Junctions Block EMT

#### 3.2.1. Organization and Function of Tight Junctions (TJ)

Tight junctions form the apical-most junctional complex in epithelial cells and play several essential structural roles [73]. First, they form a seal between cells that selectively regulates exchange of ions, macromolecules, and immune cells between the apical lumen and basolateral space. TJ mediate the blood-brain, blood-retinal, and blood-testis barriers. Second, they form a circumcellular diffusion barrier in the plasma membrane that results in separate apical and basolateral domains. Thus, integrins that bind to basal lamina cannot diffuse to the luminal face of the cell. How this apico-basal polarity is established will be covered in the next section. Third, TJ link adhesion molecules to an intracellular scaffold that anchors cytoskeletal elements and multiple signaling molecules that regulate cell proliferation and differentiation.

**Figure 2:**
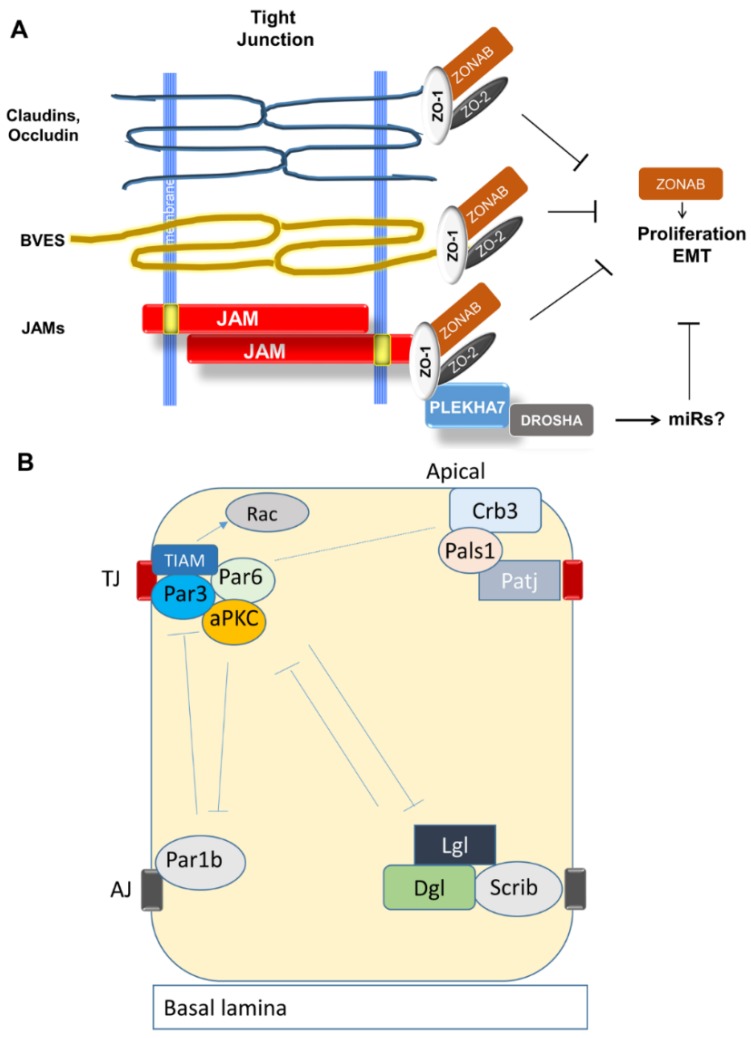
Tight Junctions. (**A**) A simple representation of signaling events at Tight Junctions showing the factors discussed in this review. For simplicity, adhesion molecules are represented as monomers. All interact independently with ZO-1, which sequesters proliferative transcription factor ZONAB. ZO-2 independently inhibits activity of other transcription factors. ZO-1 also binds PLEKHA7 to TJ (tight junction). By analogy with its role at AJ (adherens junction), PLEKHA7 may recruit miR-processing factors to TJ. (**B**) Establishment of apical-basal polarity by multiprotein complexes that specify apical and basal domains.

The cell–cell adhesion molecules that comprise TJ are illustrated in Figure 2. Occludin and claudins are tetraspanins that form very tight associations with counterparts on adjacent cells. BVES is a more recently discovered trispanin, and the Junctional Adhesion Molecules (JAMs) are type I proteins with Ig-like ectodomains. All interact with the scaffold proteins ZO-1 and ZO-2 [73].

#### 3.2.2. Establishment of Apical-Basal Polarity and Formation of TJ

During growth and development, cell–cell junction formation and maturation proceeds in stages. Following formation of stable intercellular contacts at AJ, cells must establish apico-basal polarity to segregate basal adhesive from luminal cellular functions. The nascent AJ provides positional cues, as described below.

Apico-basal polarity is determined by interactions among three complexes: Par6, Crumbs, and Scribble [74]; Figure 2B). The Par6 complex consists of three proteins, Par6, Par3, and aPKC. Crumbs consists of Crb3, Pals1, and Patj. Scribble consists of Scrib, Lgl, and Dlg. The Par6 complex localizes to the apical membrane through Par3 which also binds to the TJ protein Jam-A [75]. Crumbs binds to TJ by claudin-1 interaction with Patj [76]. In addition, the Par6 and Crumbs complexes interact with one another by Pals1 binding to Par6. The Scribble complex defines the basolateral membrane. This depends on prior formation of a nascent AJ by E-cadherin [77]. In turn, Scribble actively excludes Crumbs and Par6 from basal membranes [78]. Another AJ-associated protein, Par1b, also helps to specify the basolateral membrane by excluding the Par6 complex. The apical complexes reciprocate by excluding Scribble and Par1b from apical membranes. For a detailed review of this process, see Chatterjee [74].

The sequence of events between polarity establishment and TJ maturation is yet to be fully defined, but some clues have emerged. Chen and Macara [79] found that Par3 is required for TJ formation. Knockdown of Par3 in MDCKII cells disrupted TJ, while depletion of Par6 and aPKC did not. This effect was apparently mediated by activation of Rac. The Rac exchange factor TIAM was found to associate with Par3, and TIAM knockdown rescued TJ formation. Similarly, Suzuki *et al.* found that aPKC was not required for recruitment of claudins or occludin to cell–cell contact zones but was required for tight barrier function [80]. Others have explored the roles of ZO-1 and ZO-2. Tsukita *et al.* found that depletion of both disrupted TJ formation and AJ maturation into a continuous adhesion belt [81]. Fanning proposed that these proteins serve as a scaffold for assembly of the Apical Junctional Complex [82].

Polarity and TJ formation are actively modulated by one of the principal drivers of EMT, TGFβ. Ligation of TGFβ receptor II leads to phosphorylation of Par6 and consequent activation of ubiquitin ligase Smurf1 which causes degradation of RhoA and disassembly of TJ [83]. This action by Smurf1 regulates the local level of RhoA in lamellipodia and filopodia; RhoA in turn controls cell motility by regulating the interconversion of globular and filamentous actin [84]. The TGFβ receptor co-localizes and interacts with occludin at TJ, which presumably expedites EMT [85]. The source of TGFβ may be adjacent stromal, immune, or tumor cells [86]. Thus, autocrine signaling by a tumor cell could antagonize TJ formation by this pathway.

#### 3.2.3. Claudins

Claudins are the most diverse component of TJs. First discovered by Tsukita’s lab in the late 90’s, 24 family members have been reported to date [87,88,89]. Claudins are 20–27 kD transmembrane proteins with four transmembrane segments. Most have a cytoplasmic tail that binds to the PDZ domains of the Zonula Occludens family, ZO-1, -2, and -3 [90]. As an essential part of TJs, claudin proteins play critical roles in maintaining cell–cell integrity and in regulating paracellular ion transport [91].

The expression of some claudins and other TJ proteins is repressed by mesenchymal transcription factors in the same way that E*-*cadherin is [92]. This observation led to the naïve expectation that claudins should be uniformly downregulated in cancers, especially those associated with dedifferentiation. However, reality has proven more nuanced. There is now abundant evidence for aberrant expression or disrupted cellular localization of claudin family members during cancer progression and metastasis, but their expression patterns in cancer are highly variable and functional tests are inconsistent. A particular claudin may be tumor-suppressive in one context but promote tumor progression and/or metastasis in another (Table 1). The literature on this subject is vast. Here we consider the behavior of a few claudins that can be considered representative.

Claudin-1 was the first discovered and was found to be required for barrier function. Yet it is upregulated in colon cancer, and its expression is unexpectedly promoted by β-catenin/TCF [94]. In IHC of patient biopsies, Dhawan *et al.* observed a marked upregulation of claudin-1 and displacement from the cell membrane to the cytoplasm and nucleus with tumor progression [95]. Nuclear localization was never observed in normal colonic mucosae and rarely in primary tumors. However, 23% of lymph node metastases and 58% of liver metastases had nuclear claudin-1, while membranous expression was reduced in prevalence and intensity. Ectopic expression of claudin-1 in the primary adenocarcinoma cell line SW480 resulted in nuclear localization of the overexpressed protein and correlated with an increase in cytosolic β-catenin, increased vimentin, activation of MMP2 and MMP9, and other changes consistent with EMT [95]. On the other hand, knockdown of claudin-1 in the metastatic cell line SW620 caused MET-like changes such as restoration of β-catenin and E-cadherin to the cell membrane from the cytosol and anoikis sensitivity. Accordingly, claudin-1 ectopic expression promoted liver metastasis of SW480, and knockdown reduced liver metastasis by SW620 xenografts.

In melanoma, Leotlela *et al.* made similar observations [96]. Claudin-1 was upregulated in dermal metastases and frequently displaced to the nucleus. Melanoma cell lines had dramatic upregulation of claudin-1, but transepithelial electrical resistance tests revealed no remaining barrier function. Ectopic expression upregulated MMP2 and migration in wound assays, while knockdown in a high claudin-1 cell line reversed these effects. These authors further determined that claudin-1 expression in these cells was partly driven by protein kinase C. On the other hand, low expression of claudin-1 in lung adenocarcinoma was an indicator of increased metastasis, and knockdown enhanced invasiveness [93]. In breast cancer, the picture is equally mixed and the paradox has still not been resolved. A comprehensive review was recently published by Zhou *et al.* who showed that claudin-1 behavior varies by subtype [97]. These results suggest that claudin-1 has functions beyond TJ integrity and may have different tissue-specific and tumor-specific roles, reminiscent of E-cadherin.

Other claudins present an equally mixed picture in breast and other solid tumors. Osanai *et al.* reported that decreased expression of claudin-6 in breast cancer cells induced the activity of MMPs and promoted cancer cell migration, invasion, and anoikis-resistance [104]. Similarly, low claudin-7 correlated with a high histological grade of both ductal carcinoma *in situ* and invasive ductal carcinoma of the breast [105]. Loss of claudin-7 promoted tumor cell dissemination and increased metastatic potential. On the other hand, Philip *et al.* reported that claudin-7 promoted EMT in colorectal cancer cell lines HT29 and SW948 [106]. They demonstrated that claudin-7 recruited EpCAM to claudin-7-associated TACE and presenilin2, which then cleaved EpCAM. The cleaved EpCAM promoted EMT-associated transcription factor expression. If claudin-7 was knocked down in these cells, their migratory and invasive potential was impaired.

Martin *et al.* reported that the expression level of claudin-16 was lower in patients with poorer prognosis (with metastasis, recurrence, or mortality). Re-introduction of claudin-16 into MDA-MB-231 breast cancer cells increased junction formation and reduced aggressiveness and motility [107].

On the other hand, claudin-2 expression predicts high risk of liver cancer recurrence and poor prognosis of breast cancer [98]. Accordingly, ectopic expression of claudin-2 in MDCK-I cells that lack its expression caused a twentyfold drop in TER, suggesting that claudin-2 disrupts barrier function [99]. Similarly, claudin-3 and -4 have also been shown to be elevated in some breast cancers and correlate with invasiveness [100,101,102]. A high level of claudin-5 has been shown to promote the motility of triple negative breast cancer cell line MDA-MB-231 and reduce the survival of breast cancer patients [103].

These investigations revealed complex roles for claudins in epithelial homeostasis and tumor progression, suggesting that cancer cells find greater advantage in disrupting claudin signaling than eliminating it. The inability to form a generalized conclusion about claudins in cancer perhaps reflects the heterogeneity of cancer growth and invasion algorithms. Proteomic studies of claudin-containing complexes may resolve these conflicts, as described for E-cadherin.

#### 3.2.4. Occludin

Occludin presents a simpler picture. Like E-cadherin, it is downregulated by EMT and in claudin-low breast cancers [16]. Other expression studies confirm the downregulation of occludin protein and mRNA in metastatic breast tumors [108]. Consistent with a tumor-suppressive role, Osani *et al.* found that occludin is epigenetically silenced in breast cancer and that its loss promotes metastasis [109]. Ectopic expression sensitized HeLa cells to apoptosis and inhibited migration and tumorigenesis. These effects depended on the *C*-terminal cytoplasmic tail that interacts with ZO-1. This apoptosis-sensitizing role was also reported by Beeman *et al.* who found that occludin sensitized MEC to apoptosis when claudins were disrupted with a peptide mimetic [110]. Deletion of occludin prevented this effect. Later studies indicated a role for this function in cell extrusion [111]. Presumably tumors have no use for this function.

#### 3.2.5. Blood Vessel Epicardial Substance (BVES)

Blood Vessel Epicardial Substance (BVES) is a more recently discovered TJ trispanin structural protein that is likewise frequently downregulated during tumor progression by promoter hypermethylation and has properties of a tumor-suppressor [112,113]. Ectopic expression of a terminally truncated dominant negative mutant of BVES in human corneal epithelial cells and colon cancer cells promoted an EMT phenotype as well as upregulating RhoA and the proliferative transcription factor ZONAB. In contrast, ectopic expression of wildtype BVES reversed those effects. These results suggested an essential role for integral protein BVES in inhibiting EMT.

#### 3.2.6. Junctional Adhesion Molecules (JAMs)

Junctional Adhesion Molecules (JAMs) are small Ig domain-containing proteins expressed at TJs of epithelial and endothelial cells but also in various leukocytes [114]. Tian *et al.* reported that upregulation of JAM-A led to EMT in human nasopharyngeal cancer cells via activation of the phosphoinositide 3-kinase (PI3K) pathway, while silencing of endogenous JAM-A or treating with PI3K inhibitor reversed EMT [115]. Moreover, the authors showed a positive correlation between JAM-A expression and poor prognosis of nasopharyngeal cancer patients, suggesting a therapeutic potential for inhibiting JAM-A in these patients [115]. Others reported a tumor-promoting role for JAM-A in breast cancer [116]. However, Gotte *et al.* found that JAM-A had an anti-migratory effect in breast cancer cells [117], and others found similar results [118]. Differences in genetic background make these results difficult to interpret, as availability of adaptor and signaling molecules may vary from one cell line to the next.

#### 3.2.7. TJ Adaptor and Regulatory Molecules

Regulatory molecules at TJs play a more straightforward role in epithelial differentiation and EMT. ZO-1 and -2 are adaptor proteins that link the cytoplasmic tails of adhesion proteins to structural and signaling molecules. One of the functions of TJs is to arrest proliferative signaling by sequestering the transcription factor ZONAB. Binding ZONAB is the job of ZO-1, while ZO-2 traffics to the nucleus and downregulates AP-1-target genes such as cyclin D1 [119,120,121]. However, there is evidence that this circuit also contributes to epithelial differentiation. Tsukita’s lab developed epithelial cells lacking ZO-1 and ZO-2 [81]. The cells were devoid of TJs and deficient in the ability to activate Rac and assemble mature belt-like AJs. Georgiadis and co-workers found that ZO-1 knockdown or ZONAB over-expression resulted in increased proliferation of retinal pigment epithelium (RPE) *in vivo*, with EMT-like changes, suggesting a critical role of ZO-1 in maintaining differentiation and homeostasis of the RPE monolayer [122]. These results suggest there is cross-talk between anti-proliferative and differentiative signaling pathways.

The latest development in this story is that the apical AJ protein PLEKHA7 has also been found at TJs and shown to regulate their assembly [123]. This implies that TJs may affect differentiative signaling by regulating processing of a set of microRNAs, perhaps overlapping with those regulated by AJs. Whether a TJ protein associates with PLEKHA7 may determine whether it favors MET or EMT and thus its effect in a particular tumor cell.

## 4. Targeting EMT Therapeutically

In light of the mounting evidence revealing EMT as a central element for drug resistance and tumor metastasis in various types of cancer, targeting the EMT process for anti-cancer drug development has attracted great interest. Pharmacological targeting of crucial signal transduction pathways offers several opportunities to inhibit the induction of EMT. A good example is the TGF-β/Smad pathway, which is a principal inducer of EMT. TGF-β was first identified as a pro-EMT signaling inducer by Miettinen *et al.* in 1994 [124]. Since then, the role of TGF-β in promoting physiological and pathological EMT has been well described [125,126]. Inhibition of the TGF-β receptor kinase using its specific inhibitor SB431542 blocks TGF-β-induced EMT in pancreatic cancer cells [127].

Another example is transcription factor STAT3 (signal transducer and activator of transcription 3), which has been shown to have critical roles in EMT induction in a variety of human cancers. Sorafenib, a potent multi-kinase inhibitor, suppressed TGF-β-mediated EMT through the suppression of STAT3 phosphorylation in mouse hepatocytes and AML12 [128].

The various cellular components of the tumor microenvironment, including fibroblasts, endothelial cells, and immune cells, are important sources of soluble factors triggering EMT, including cytokines and growth factors. Therapies targeting these factors or corresponding receptors in or on cancer cells represent another strategy for preventing EMT. Lo *et al.* demonstrated that EGF receptor kinase inhibitor AG1478 can suppress EMT induction mediated by EGF in human breast cancer cells [129]. However, due to the multiple factors and signaling pathways involved in EMT and the complexity of the tumor microenvironment, it is possible that targeting a single factor or pathway may not suffice to fully reverse EMT.

EMT is accompanied by acquisition of a set of mesenchymal markers, including vimentin, N-cadherin, fibronectin, and MMPs, that are associated with increased drug resistance, cell migration, and invasion capability. In effect, N-cadherin is a cell surface marker for the most pernicious subset of cancer cells and this can be exploited therapeutically. In prostate cancer, N-cadherin marks androgen-independent, invasive tumors [130]. Targeting N-cadherin using a monoclonal antibody blocked prostate cancer invasion and metastasis in xenografts and even caused tumor regression at higher dosages.

Since EMT induces the formation of breast cancer stem cells [11], artificially induced EMT could be used to generate large numbers of CSCs and screen for agents that are specifically toxic to them. A pharmaceutical library yielded several compounds that affect transmembrane ion currents. The most potent was a potassium ionophore called salinomycin that was specifically toxic to the CD44HIGH/CD24LOW stem cell subpopulation. The ability to selectively eliminate CSCs in combination with agents that target the proliferative non-CSCs would be expected to greatly curtail cancer relapse [131,132].

Another selective inhibitor for breast cancer stem cells is metformin, a first-line drug for diabetes. Heather *et al.* reported that a low dose of metformin selectively targeted this subpopulation [133]. Combining it with conventional chemotherapeutic doxorubicin killed both stem cells and non-stem cells *in vitro*. *In vivo*, this combination more effectively reduced tumor mass and inhibited tumor relapse than either drug alone. Further investigation demonstrated that metformin selectively blocked the nuclear translocation of NF-kB and phosphorylation of STAT3 in the stem cell population, while non-stem cells were less sensitive [134]. The action of metformin involved the inhibition of inflammatory responses. If metformin was administrated after the initial stimulation of inflammatory loop, it had no effect in inhibiting transformation. Since many inflammatory factors are also EMT stimulators, metformin is probably able to inhibit cancer cell EMT by reducing EMT-promoting factors in the tumor microenvironment.

Finally, the linkage of EMT–MET signaling to microRNA networks opens another new therapeutic avenue. For example, AC1MMYR2, a miR-21 inhibitor, suppressed EMT and inhibited invasion in several cancer types including breast cancer [135]. However, pharmacologically, miR mimetics have been more successful than miR inhibitors. Thus, the finding that the PLEKHA7 microprocessing complex activates miR30b, an inhibitor of EMT and proliferation, offers the possibility of inhibiting proliferation, migration/invasion, and CSCs with a single targeted agent. Given the immunoresistance conferred by EMT, such an agent could also improve the efficacy and range of application of immune checkpoint inhibitors. We suggest that discovering what additional miRs are dependent on these junctional processing centers should become a priority in cancer research. These developments should bring renewed appreciation for the utility of basic research on the structure and function of cell–cell junctions in maintaining tissue homeostasis.

That said, one widely recognized limitation of any anti-EMT therapy is that metastatic cancer cells undergo MET upon reaching distant organ sites, and this step appears necessary for colony formation [28]. Moreover, circulating tumor cells typically have a mesenchymal profile, raising the possibility that anti-EMT therapies might enhance tumorigenesis by these dispersed cells [32]. Thus, extensive preclinical testing will be required in long-lived animal models. Ideally, any anti-EMT therapy would also be anti-proliferative. Hybrid miR mimetics with both properties are one possible solution to this conceptual dilemma. Sustained research on homeostatic signaling mechanisms may yield others yet dreamed of.

## 5. Conclusions

While cell-cell junctions play an integral role in suppressing tumorigenesis and progression, the literature reviewed here makes it clear that the signals that emanate from intercellular junctions are not exclusively anti-neoplastic. Cell-cell adhesion molecules such as claudins, N-cadherin, and even E-cadherin may play vital roles in cell migration. Driven by natural selection, cancer will opportunistically exploit whatever algorithms it finds in the nuclear toolbox of its tissue and cell-type of origin. This may mean shedding epithelial differentiation entirely, as in triple-negative breast cancers, and using stem-cell programs to emigrate as individual free-agents, later to invade and re-differentiate elsewhere. On the other hand, tumor cells may find growth and survival advantage in jiggering epithelial homeostatic programs to permit rapid proliferation in situ. Such cells may maintain robust junctional moorings until partial EMT or tissue disruption allows dispersal of cells singly or in small clusters that lodge in distant capillary nets and eventually give rise to new tumors. Many breast cancer and other cell lines maintain high expression of E-cadherin and other cell-cell adhesion molecules [136]. Such tumors can often be controlled initially with inhibitors of hormonal or growth factor signaling but eventually become resistant. A better understanding of how such tumors subvert homeostatic signaling could result in new combinatorial strategies to suppress relapse while minimizing toxicity to normal tissues.

## Figures and Tables

**Table 1:** Putative roles of claudins in various cancers.

Type of Claudin	Tumor Suppressor	Oncogene
Claudin-1	Lung cancer [93]	Colon cancer [94,95]
	-	Melanoma [96]
	Breast cancer [97]	Breast cancer [97]
Claudin-2	-	Breast cancer [98,99]
Claudin-3	-	Breast cancer [100,101]
Claudin-4	-	Breast cancer [100,101,102]
Claudin-5	-	Breast cancer [102,103]
Claudin-6	Breast cancer [104]	-
Claudin-7	Breast cancer [105]	Colorectal cancer [106]
Claudin-16	Breast cancer [107]	-
